# An Unusual Etiology of Aortic Insufficiency: A Case Report

**DOI:** 10.7759/cureus.30915

**Published:** 2022-10-31

**Authors:** Robert Libera, Ibiyemi Oke, Smit Shah, Shefali Amin, Agnieszka Mochon

**Affiliations:** 1 Internal Medical, Reading Hospital/Tower Health, West Reading, USA; 2 Internal Medicine, Reading Hospital/Tower Health, West Reading, USA; 3 Cardiology, Reading Hospital/Tower Health, West Reading, USA; 4 Internal Medicine, Reading Hosptial/Tower Health, West Reading, USA

**Keywords:** echocardiography, valvular regurgitation, valvular heart disease, aortic insufficiency, aortic valve, leaflet retraction

## Abstract

Identifying the etiology of aortic insufficiency (AI) is essential in the management of the patient with valvular heart disease. We report the case of a 34-year-old male who presented with New York Heart Association (NYHA) class IV symptoms. The physical exam was consistent with AI, which was confirmed on echocardiography. Interestingly, the trileaflet aortic valve (TAV) was comprised of three retracted cusps, a rarely cited anatomic abnormality yielding AI. The patient underwent an uncomplicated aortic valve replacement (AVR).

## Introduction

Aortic insufficiency (AI) presents with a classical array of symptoms and physical exam findings; however, the precipitating event or associated condition causing this presentation is often far less clear. While less common than other valvular pathologies, AI has a reported prevalence of 13% and 8.5% for males and females, respectively, based on the population studied in the Framingham Study [1]. In addition to gender discrepancy, AI is also more frequently observed with advanced age [2].

In AI, malcoaptation of the aortic valve (AV) leaflets results in left ventricular (LV) volume overload as inappropriate regurgitant blood volume combines with effective stroke volume [2,3]. Physiologically, AI is distinguished from other regurgitant states, such as mitral regurgitation, by its increase in afterload in addition to increased preload [3]. Chronically, this leads to eccentric hypertrophy of the LV and decreased ionotropic function [2]. Clinically, patients classically present with wide pulse pressure, bounding pulses, and a loud diastolic murmur [4]. Early recognition and appropriate management of AI are imperative, considering the increased morbidity and mortality of severe AI [5]. In this report, we present a case of AI secondary to a rare congenital leaflet abnormality and discuss etiologies to be considered when presented with undifferentiated AI.

## Case presentation

A 34-year-old male with a past medical history of dilated cardiomyopathy, hypertension, and tobacco abuse presented to the emergency department complaining of progressively worsening dyspnea on exertion for three days. Over that time frame, the patient's activity tolerance decreased; shortness of breath bothered him during routine activities such as climbing stairs. The patient also described exertional non-radiating substernal chest discomfort, orthopnea, and progressive bilateral lower extremity swelling. He denied pleuritic chest pain, palpitations, lightheadedness, dizziness, or syncopal events. The patient denied excessive alcohol intake, illicit drug abuse, or over-the-counter supplements. 

The patient recalled being told that he had high blood pressure when he was about 17 years old; however, he had never taken any antihypertensives. Additionally, the patient reported he was told that his "heart was dilated" about three years prior but never followed up with a physician. Family history was negative for ischemic heart disease or cardiomyopathy.

In the emergency department, the patient's blood pressure was 184/68, heart rate was 111 beats per minute, respiratory rate was 41, and oxygen saturation was 99%. He did not require supplemental oxygen. He was afebrile. Generally, the patient was anxious and tachypneic but non-toxic appearing. On the cardiovascular exam, S1 and S2 were regular. A grade three diastolic murmur was appreciated at the left sternal border at the third intercostal space. The carotid upstroke was brisk, and palpation of the radial artery was consistent with Corrigan's pulse. There was bilateral plus one lower extremity pitting edema to the mid-shin. Neck veins were not distended. A pulmonary exam was significant for bibasilar rales.

**Figure 1:**
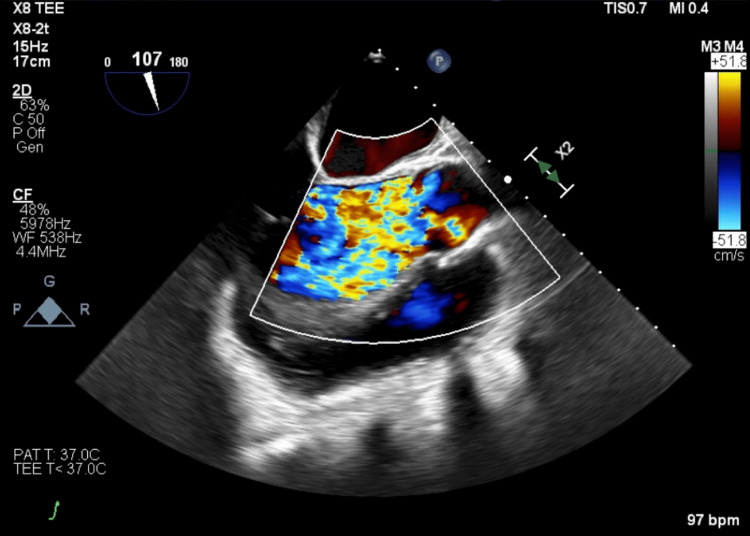
Transesophageal echocardiogram: 2D mid-esophageal long axis view at end-diastole with color-flow Doppler showing visually severe central aortic insufficiency

**Figure 2:**
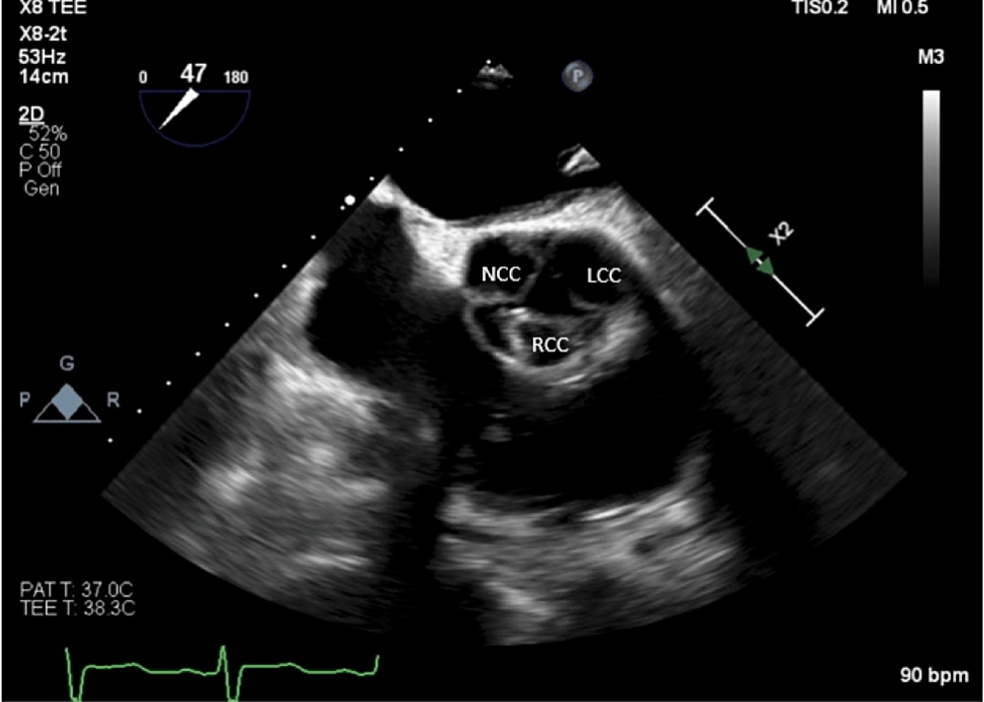
Transesophageal echocardiogram: 2D short axis aortic view at end-diastole The image shows the retraction of the tip of all three leaflets of the aortic valve, particularly the right coronary cusp. NCC - non-coronary cusp; LCC - left coronary cusp; RCC - right coronary cusp

**Figure 3:**
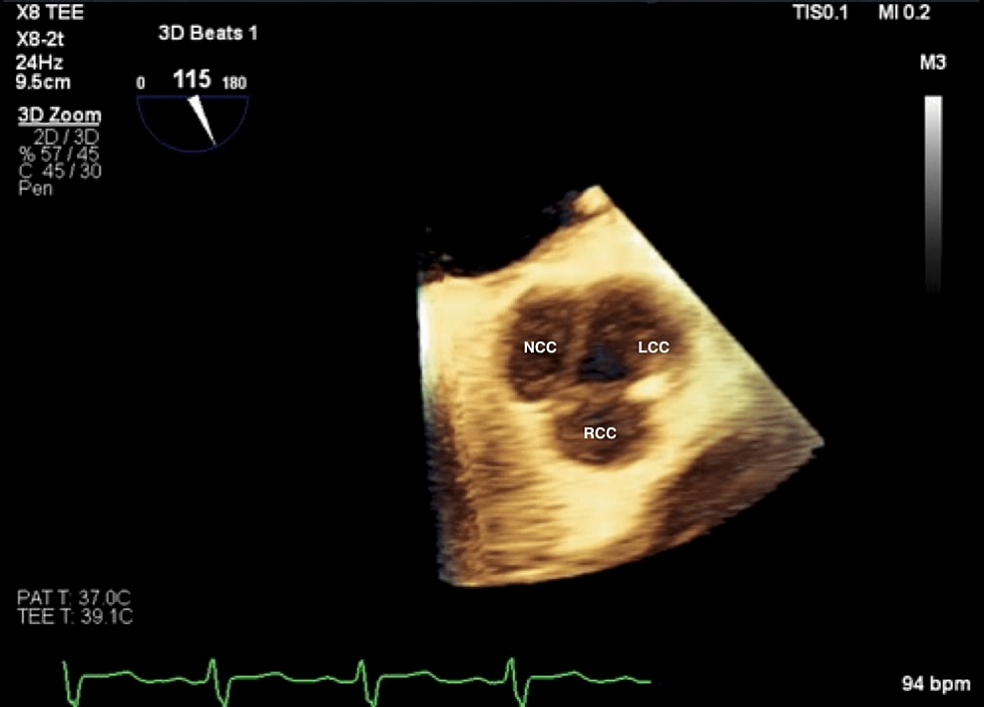
Transesophageal echocardiogram: 3D short axis aortic view at end-diastole The image shows the retraction of the tip of all three leaflets of the aortic valve, particularly the right coronary cusp. NCC - non-coronary cusp; LCC - left coronary cusp; RCC - right coronary cusp

Initial laboratory findings included brain natriuretic peptide (BNP) of 1439 and troponin of 0.14. Chest X-ray was consistent with pulmonary vascular congestion. ECG revealed sinus tachycardia and left atrial enlargement. A transthoracic echocardiogram demonstrated a markedly dilated left ventricle which was severely dysfunctional in a global fashion. The ejection fraction was 20%. Left ventricular diastolic and systolic diameters were 9.4 cm and 8.4 cm, respectively. The aortic valve appeared to be trileaflet, and regurgitant stroke volume was estimated to be greater than 50%, indicating severe aortic insufficiency (Figure 1). A transesophageal echocardiogram confirmed a trileaflet aortic valve; however, all three leaflets appeared retracted, especially at the tips, with the right coronary cusp most severely affected (Figures 2, 3). Left-sided cardiac catheterization demonstrated patent coronaries, and a dobutamine stress test revealed augmentation of cardiac output and ejection fraction.

Prior to aortic valve replacement (AVR), the patient was medically managed with diuretics and afterload reduction with lisinopril. Despite medical management, the patient still experienced orthopnea and dyspnea with minimal activity such as during ambulation to the bathroom. Due to the refractory nature of his symptoms and the severity of LV dilation, cardiothoracic surgery was consulted. Intraoperatively, all three cusps were confirmed to be retracted, and the valve was not amenable to repair. In part, the replacement was preferred over repair, considering the patient's young age. The patient underwent bioprosthetic AVR, given his history of medication nonadherence and follow-up inconsistencies. Following an uncomplicated post-operative course, the patient was discharged on aspirin, metoprolol succinate, and lisinopril for his cardiomyopathy (sacubitril-valsartan was cost prohibitive) and had a life vest on prior to discharge.

## Discussion

AI can be categorized by chronicity and by affected structures such as a leaflet or an aortic root abnormality [6]. Rheumatic disease remains the most common cause of AI in developing countries, while congenital abnormalities, particularly bicuspid AV, are the leading cause of AI in Western Europe and North America [2]. AI has a myriad of disease associations that are important to consider. Some autoimmune diseases, vasculitides, congenital syndromes, and connective tissue disorders can have cardiac manifestations, including AI [6].

In their retrospective review of patients with native valve AI who underwent aortic valve replacement, Yang et al. found that most patients had multiple contributory factors. In the minority of patients with isolated causes, leaflet restriction/retraction was responsible for 9% of occurrences [7]. It is unknown what percentage of this subgroup suffered from trileaflet retraction, as was the case in our patient.

Once the diagnosis of AI has been made, management can be guided by parameters observed on echocardiography and symptomatology as outlined in the 2020 American College of Cardiology (ACC)/American Heart Association (AHA) Guideline for the Management of Valvular Heart Disease [8]. In our case, the patient met the criteria for severe AI, which is a class I indication for AVR.

## Conclusions

In this report, we present a rare cause of AI, trileaflet retraction of the native AV. Incidence, genetic susceptibility, and associated medical conditions related to leaflet retraction are incompletely understood. When confronted with AI of undetermined etiology, we encourage consideration of trileaflet retraction in the differential. Reports such as this one can help guide future investigations of leaflet retraction with the aim of identifying individuals at risk of AI from this rare cause.
